# Commentary: Association between life’s essential 8 and male biochemical androgen deficiency: evidence from NHANES 2013-2016

**DOI:** 10.3389/fendo.2024.1462809

**Published:** 2024-09-03

**Authors:** Fei Zhang, Sheng Li

**Affiliations:** ^1^ Department of Urology Surgery, Ningbo No.2 Hospital, Ningbo, Zhejiang, China; ^2^ Department of Anorectal Surgery, Ningbo No.2 Hospital, Ningbo, Zhejiang, China

**Keywords:** life’s essential 8, biochemical androgen deficiency, endocrine function, reproduction, NHANES

## Introduction

We were intrigued to read the paper by Huang et al. titled “Association between Life’s Essential 8 and Male Biochemical Androgen Deficiency: Evidence from NHANES 2013–2016.” (1) This study innovatively explores the relationship between Life’s Essential 8 (LE8) (2) and male biochemical androgen deficiency (MBAD), as well as total testosterone (TT) levels in the U.S. population using data from the National Health and Nutrition Examination Survey (NHANES) from 2013 to 2016. The authors utilized a large, nationally representative sample and rigorous data collection methods, employing advanced statistical techniques such as multivariate analysis, propensity score matching (PSM), and weighted regression to control for confounding variables. They concluded that higher LE8 scores are independently associated with a lower likelihood of MBAD and higher TT levels. This study innovatively explores the relationship between LE8 and MBAD, suggesting routine monitoring of LE8 as an important measure for preventing and managing MBAD. However, we still have some questions regarding this study.

## Covariate selection

Firstly, the study includes important covariates such as age, race, education level, marital status, poverty rate, sample collection timing, and self-reported cardiovascular disease (CVD), which is commendable. However, biochemical markers that may affect testosterone levels, such as uric acid (UA), total cholesterol (TC), and high-density lipoprotein (HDL) (3), should also be considered as potential covariates.

### Outcome variable selection

In this study, the authors designated MBAD status as the outcome variable, with MBAD being defined as a serum testosterone level <300 ng/dL, and a testosterone threshold was identified as the only inclusion criterion for MBAD. Serum testosterone levels in NHANES were measured using precise isotope dilution liquid chromatography and tandem mass spectrometry to measure serum testosterone values at a single time point in the morning, afternoon, or evening. This involves two problems. One is that serum testosterone concentrations show a diurnal variation, with peak testosterone levels between 6 a.m. and 7 a.m. and nadir levels between 7 p.m. and 8 p.m (4). Serum testosterone levels can fall by 50% or more over the course of a day, and aging reduces the magnitude of this diurnal variation (5). Despite the diminished circadian rhythm in older men, a large proportion of men aged 65-80 years with low afternoon serum testosterone concentrations have normal testosterone concentrations in the morning (6), and glucose and food intake suppress testosterone concentrations (7). This resulted in a portion of subjects with normal testosterone levels who underwent blood draws at time points when testosterone production was low, or those with low testosterone levels after eating, being incorrectly classified in the MBAD group. Second, in a community-based, multiethnic cohort of middle-aged and older men, the day-to-day variability in serum testosterone concentrations was so great that a single testosterone measurement was insufficient to characterize an individual’s testosterone concentration. Brambilla et al.’s study (8) noted that 30% of men whose initial testosterone concentration was in the hypogonadal range had a normal testosterone concentration on repeat measurements. Therefore at least two testosterone measurements are needed to confidently diagnose MBAD (9). Both of these points largely confound the outcome variables and affect the results of the overall study.

### Predictive ability assessment

The LE8 score includes four health behaviors (diet, physical activity, nicotine exposure, and sleep duration) and four health factors (BMI, non-HDL cholesterol, blood glucose, and blood pressure). The authors suggest that using LE8 scores as an assessment tool can reduce the burden of MBAD. However, the authors did not evaluate the predictive ability of LE8 based on the occupational characteristics of the subjects. We encourage the authors to compare the predictive ability of LE8 with one or more of these factors, which would help assess whether LE8 can be more widely used as a better predictive tool.

## Discussion

In conclusion, this is an intriguing study, but we hope the authors address our questions to make the paper more accurate and comprehensive, thereby aiding readers in better understanding the relationship between LE8 and MBAD.
